# Resonance optimization of polychromatic light in disordered structures

**DOI:** 10.1038/s41598-017-08635-1

**Published:** 2017-08-14

**Authors:** Hongwei Yin, Adenowo Gbadebo, Elena G. Turitsyna, Sergei K. Turitsyn

**Affiliations:** 1 College of Physics and Microelectronic Science, Hunan University, Changsha, 410082 China; 2 Aston Institute of Photonic Technologies, Aston University, Aston Triangle, Birmingham B4 7 ET UK

## Abstract

Disorder offers rich possibilities for manipulating the phase and intensity of light and designing photonic devices for various applications including random lasers, light storage, and speckle-free imaging. Disorder-based optical systems can be implemented in one-dimensional structures based on random or pseudo-random alternating layers with different refractive indices. Such structures can be treated as sequences of scatterers, in which spatial light localization is characterized by random sets of spectral transmission resonances, each accompanied by a relatively high-intensity concentration. The control and manipulation of resonances is the key element in designing disorder-based photonic systems. In this work, we introduce a method of controlling disorder-induced resonances by using the established non-trivial interconnection between the symmetry of bi-directional light propagation properties and the features of the resonant transmissions. Considering a fiber with resonant Bragg gratings as an example, the mechanism of enhancing or suppressing the resonant transmission of polychromatic light and the effectiveness of the method have been demonstrated both theoretically and experimentally. The proposed algorithm of controlling disorder-induced resonances is general and applicable to classical waves and quantum particles, for disordered systems both with and without gain.

## Introduction

Design and operation of a variety of optical devices, such as lasers, switches, modulators of light, and many others, require efficient algorithms for controlling and manipulating radiation-matter interactions. Nowadays, the majority of these methods rely upon periodic photonic structures (for example, gratings or photonic crystals) whose band structures enable appropriate light harnessing. However, the random deviations from periodicity that are inevitably present in any manufactured sample often create a serious obstacle in the practical use of periodic structures, or limit their application. An alternative to pursue perfect periodicity is to take an opposite extreme: the fabrication of highly disordered structures that could be harnessed to create tunable resonant elements. The design of photonic devices based on disorder has attracted a great deal of attention in recent years^1–8^. Random variations of the refractive index can occur due to inherent material properties, for example the scattering of light on the irregularities of a medium, or they can be “engineered”^9^. A well-known example of a disorder-based device is the random laser^10, 11^. The concept of photon localization in disordered optical mediums connects fundamental theoretical physics and optical engineering, creating new possibilities for the spectral and spatial control of light.

In particular, one-dimensional (1D) random structures (for example, randomly layered single-mode waveguides and fibers) combine the perfect directionality of light along the system with unique spectral and transport properties that, for many applications, offer advantages over properties of periodic structures. In a closed, isolated 1D disordered system, all eigenstates (normal modes) are exponentially localized. When a sample is open, that is, it is coupled to the environment due to the finite transparency of the edges, it is characterized by quasi-normal modes, which can be found as the field distributions satisfying outgoing boundary conditions^12^. The eigenfrequencies of the quasi-normal modes are complex, and their imaginary parts are the inverses of the corresponding lifetimes. In the localized regime, the transmission spectrum consists of wide “gaps” where the transparency is exponentially small with relatively narrow resonances - maxima of the transmission coefficients at frequencies that are equal to the real parts of the eigenvalues of the corresponding quasi-normal modes. At each resonant frequency, a random 1D configuration can be considered as an open resonator with a high quality factor. In stark distinction to a regular resonator whose modes occupy all inner space, in a 1D random structure, quasi-normal modes are spatially extended and overlap, making individual mode selection extremely challenging^13^. Since the resonances are sharp and sensitive to small changes in the parameters of the configuration, one can switch a sample from reflection to transmission, or tune the emission of a source located inside the sample by external actions, for example illuminating it with electromagnetic radiation that changes the dielectric constant of the material due to nonlinear effects^14, 15^. This potentially offers functionality much richer than that in periodic photonic structures. However, a substantial problem in the practical exploitation of disorder-based structures is that their parameters are random and hard to predict. One of the major challenges in designing disorder-based photonic elements is a capability to manipulate resonances that correspond to the localized states. We would like to mention that the scheme for the construction of the random potential, elaborated in the paper, has some similarity with the approach used for modeling random dispersion management in fiber-optic systems^16, 17^.

Most of the available theoretical results deal with *ensemble averaging over a large number of random realizations*. The description of the properties of a disordered system for a *particular distribution* of scatters is a much more challenging theoretical and engineering problem. High-intensity concentration within the disordered mediums can be considered as an “effective cavity”^5, 12, 18, 19^. Characteristics of transmission resonances, for example width, are highly sensitive to the spatial position of the effective cavity in the system. To the best of our knowledge, there are no results in the literature on designing spectral resonances by controlling the position of the effective cavity in the system.

In this work, we find a link between the symmetry of the bi-directional light propagation properties and the measurable features of resonant transmission spectra of the disordered system. We apply this link to the efficient design of the transmission characteristics of multiple resonances via a disorder micro-modification.

## Results

### Resonance description and resonance optimization method

**Figure 1:**
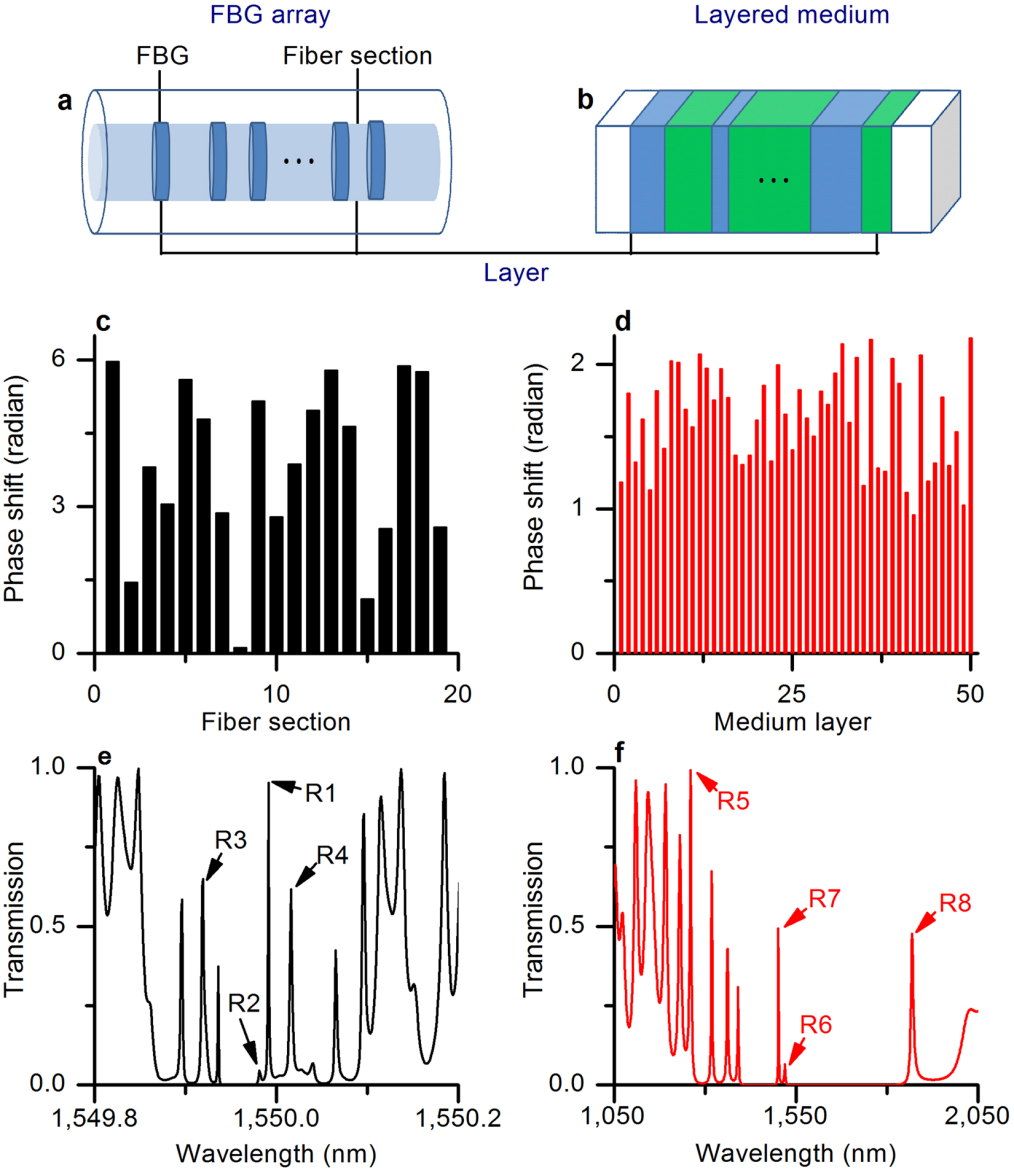
Disordered mediums and disorder-induced resonant transmissions. (**a**) FBG array; (**b**) layered medium. Random phase shifts in (**c**) the FBG array, and (**d**) the layered medium. Disorder-induced resonant transmissions in (**e**) the FBG array, and (**f**) the layered medium.

To demonstrate the principle of the proposed method of resonance optimization in 1D disordered systems, we consider two practical implementations: a fiber Bragg grating (FBG) array (Fig. 1a), and a layered dielectric medium (Fig. 1b). The first system is built of identical FBGs separated by homogeneous fiber sections of random lengths. In our simulations, we considered an FBG array formed of 20 uniform, 3-mm-long gratings (with a refractive index change of 10^−4^), with the Bragg wavelength of *λ*_*B*_ = 1,550 nm, the peak reflectivity of 0.20, and the full width at half maximum of 0.27 nm. The layered medium consisted of 50 alternating layers with the refractive indices *n*_1_ = 2.1 and *n*_2_ = 1.4, and with the central wavelength *λ*_0_ = 1,550 nm. The wavelength of the incident wave considered in the intervals was 1,549.8 < *λ* < 1,550.2 nm for the FBG array, and 1,050 < *λ* < 2,050 nm for the random layer simulations. In both cases, the disorder was introduced through the lengths of the random cavities; that is, by varying the phase shifts between incident and transmitted waves, which are assumed to be uniformly distributed from 0 to 2*π* in fibers and from 0.3*π* to 0.7*π* in randomly layered mediums.

The second system consists of alternating layers of random lengths with different refractive indices (*n*_1_ and *n*_2_). Particular examples of random distributions of the phase shifts in the FBG array and the layered medium are shown in Fig. 1c and d, respectively. The corresponding resonant transmission coefficients are shown in Fig. 1e and f. Eight resonances with transmission values R1–R8 were selected for the analysis and optimization (Fig. 1e and f).

**Figure 2:**
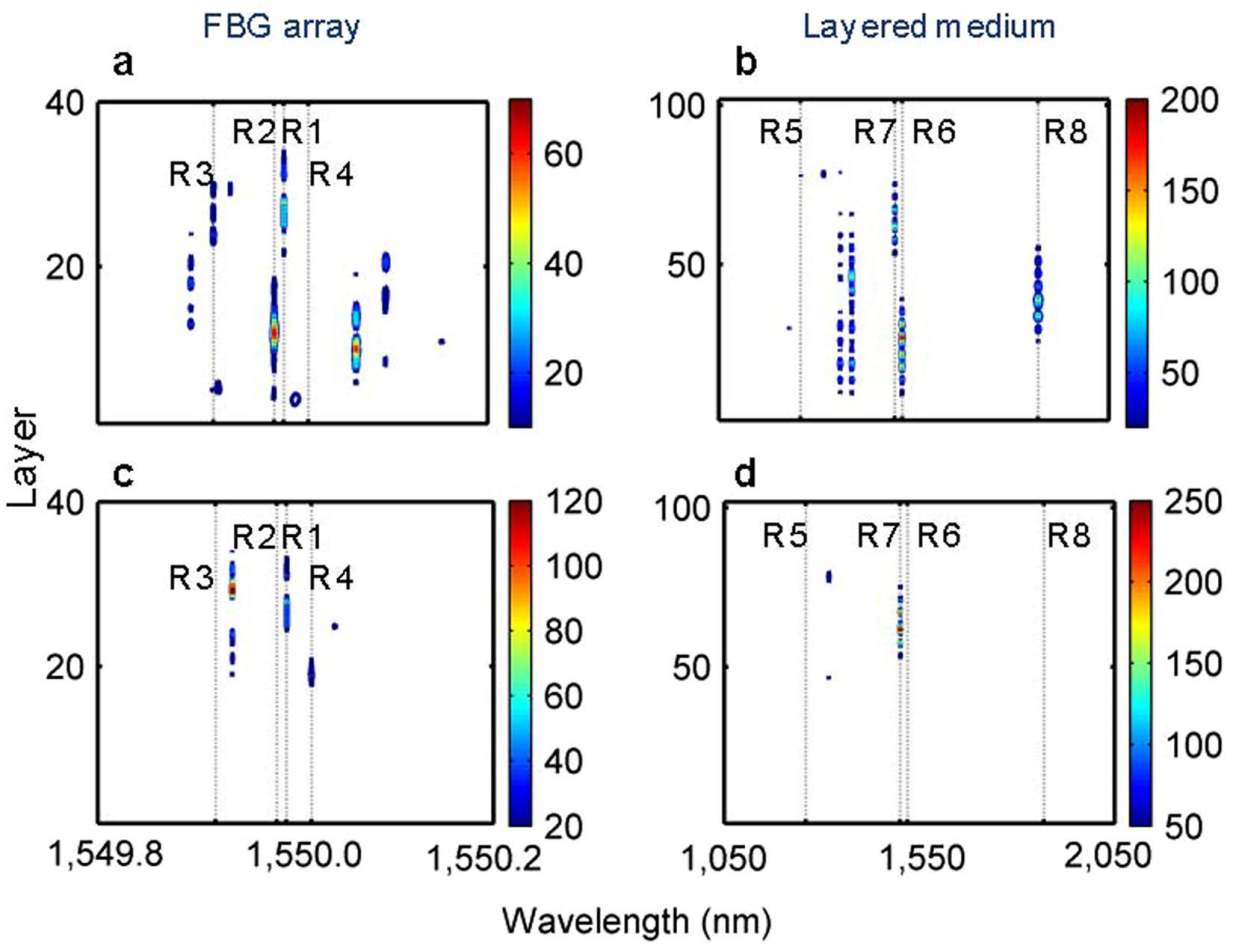
Disorder-induced spatial-frequency bi-directional high-intensity concentrations. **(a**) Left-incident intensity in the FBG array. (**b**) Left-incident intensity in the layered medium. (**c**) Right-incident intensity in the FBG array. (**d**) Right-incident intensity in the layered medium.

**Figure 3:**
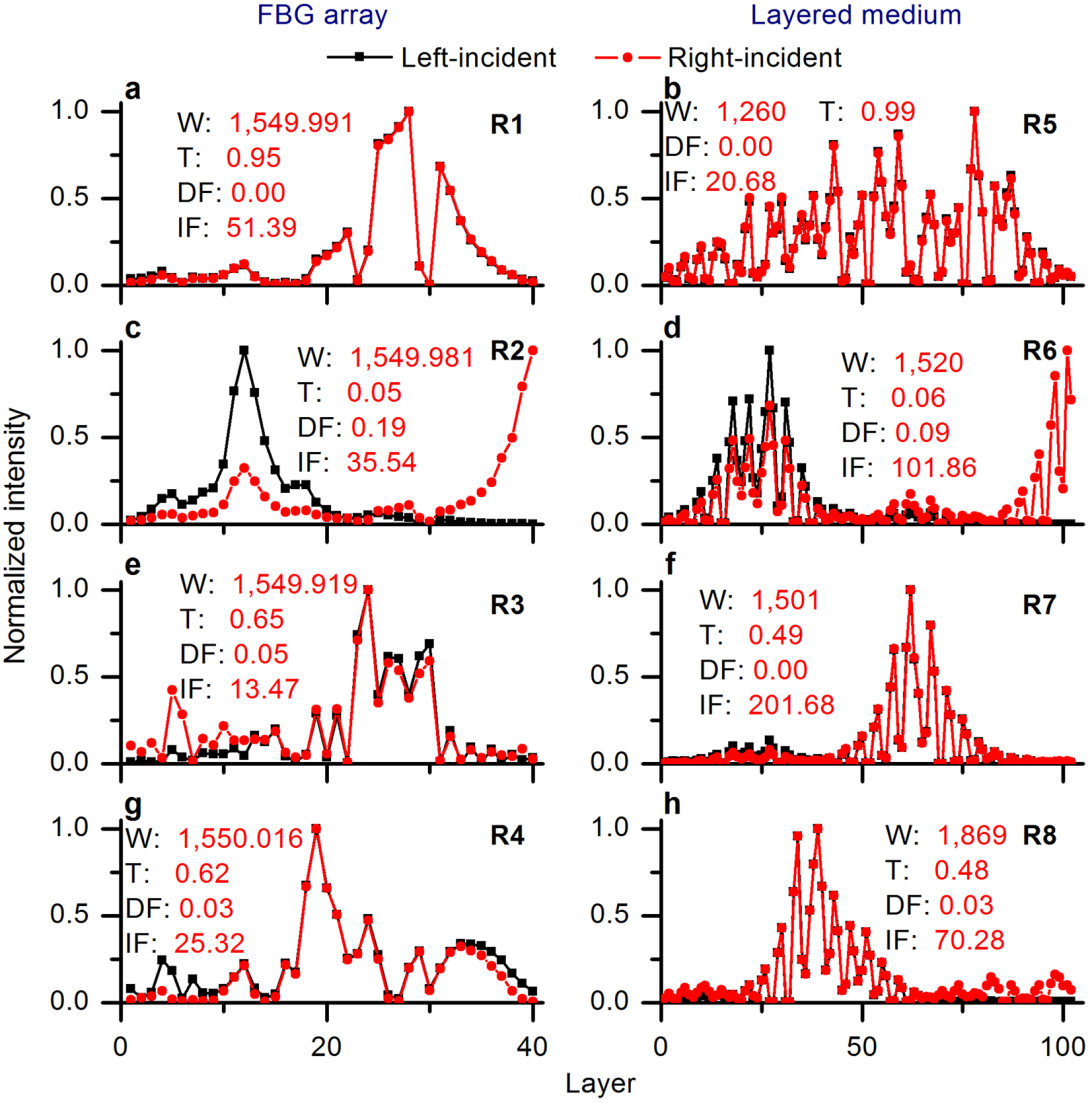
Normalized bi-directional intensity distributions in the FBG array and the layered medium. **(a**) R1. (**b**) R5. (**c**) R2. (**d**) R6. (**e**) R3. (**f**) R7. (**g**) R4. (**h**) R8. W: wavelength in nm, T: transmission, DF: deviation factor, IF: intensity factor.

The approach proposed here is based on the following simple observation: while the transmission coefficients are independent of the direction of incidence, the intensity distributions created by the left- and right-incident waves are, in general, different. This feature of the disordered 1D systems will be used in a new spatio-spectral design method. Asymmetry between left-propagating and right-propagating light also appears in the high-intensity field concentration that is another characteristic feature of 1D light localization. The spatial-frequency high-intensity field concentrations in the random FBG array and in the random layered medium are shown in Fig. 2. Figure 2 clearly demonstrates the effect of the directionality of the incident light (left- and right-incident). One can see from Fig. 2 that high-intensity concentrations always occur at the resonances of transmission (compared to Fig. 1e and d) and such high-intensity areas are sensitive to the direction of the incident light. The normalized bi-directional intensity distributions of the eight selected resonances R1–R8 are shown in Fig. 3. The normalized intensity distributions defined in this work naturally represent the effective spatial cavities associated with spectral resonances.

To quantify the asymmetry of the light propagation in the left and right directions at each wavelength, we introduce the deviation factor (DF), which is an integral (over space) characteristic of the differences between the intensities of left- and right-propagating light. Left-incident and right-incident spatial-frequency intensity distributions generated by incident monochromatic radiation with the wavelength *λ* are described by *I*_*L*_(*m*;*λ*) and *I*_*R*_(*m*;*λ*), where m is the layer number counted from the left edge of a sample, and the subscripts *L* and *R* represent the left-incident and right-incident directions, respectively. The bi-directional spatial-frequency intensity distributions can be further normalized by

$$\{\begin{array}{rcl}{\bar{I}}_{L}(m;\lambda ) & = & {I}_{L}(m;\lambda )/\,\max \,{I}_{L}(m;\lambda )\\ {\bar{I}}_{R}(m;\lambda ) & = & {I}_{R}(m;\lambda )/\,\max \,{I}_{R}(m;\lambda )\end{array}$$

where ${\bar{I}}_{L}(m;\lambda )$ and ${\bar{I}}_{R}(m;\lambda )$ are the left-incident and right-incident normalized intensity distributions, respectively. The normalized intensity distribution naturally represents the effective cavity. To characterize the localization of light in space, we introduce the intensity factor (IF), which is a maximum (over all layers) of a half of the sum of the left- and right-propagating light intensities. To quantify the asymmetry of the light propagation in the left and right directions at each wavelength, we introduce the deviation factor (DF), *β*_*dev*_, to quantitatively describe the symmetry of the bi-directional effective cavities, and the intensity factor (IF), *β*_*int*_ to quantify the bi-directional intensity concentrations.

$$\{\begin{array}{rcl}{\beta }_{dev}(\lambda ) & = & \sum _{m\mathrm{=1}}^{M}|{\bar{I}}_{L}(m;\lambda )-{\bar{I}}_{R}(m;\lambda )|/M\\ {\beta }_{int}(\lambda ) & = & {\rm{\max }}\,[{I}_{L}(m;\lambda )-{\bar{I}}_{R}(m;\lambda \mathrm{)]/2}\end{array}$$

where *M* is the total number of layers. By combining the DF and the IF we introduce the localization factor (LF), which is used as an *optimization parameter*. The integral localization properties of the sample are given by the total localization factor (TLF) obtained by summing the LF of multiple resonances: *J*(*λ*) = −log[*β*_*dev*_(*λ*) × *β*_*int*_(*λ*)]. The total localization factor (TLF) of (*λ*_1_, *λ*_2_, …, *λ*_*G*_) resonances with the wavelengths can be obtained by $J({\lambda }_{1},\ldots ,{\lambda }_{G})={\sum }_{i\mathrm{=1}}^{G}J({\lambda }_{i})$.

One can see from Figs 1, 2 and 3 that there is a clear link between the directionality of light propagation, localization, and the transmission resonances. The retrieval of some internal parameters of the disordered mediums from the externally measurable resonant transmission had been studied^18^. However, in the present work, we make a major new step and demonstrate a breakthrough possibility of the controlled localization-delocalization of random resonances achieved by exploiting the existing link between the externally measurable resonant characteristics, including spectral width and the bi-directional high-intensity concentrations.

We would like to emphasize that by using the proposed approach, it is possible to optimize both the resonant transmission and the width of multiple resonances by controlling the TLF via a disorder micro-modification. The developed resonance design scheme is based on the stochastic parallel gradient descent (SPGD) method^20^ (see Methods). The TLF is continuously ascending for localization (enhancement) resonance optimization, or descending for delocalization (suppression) resonance optimization, until the preset (desired) feature is achieved. Note that although only phase shift disorder is considered in this work, the proposed resonance optimization algorithm is readily applicable to other kinds of disorder.

### Resonance optimization of FBG array without gain - possibility to design disorder-based localization filter

**Figure 4:**
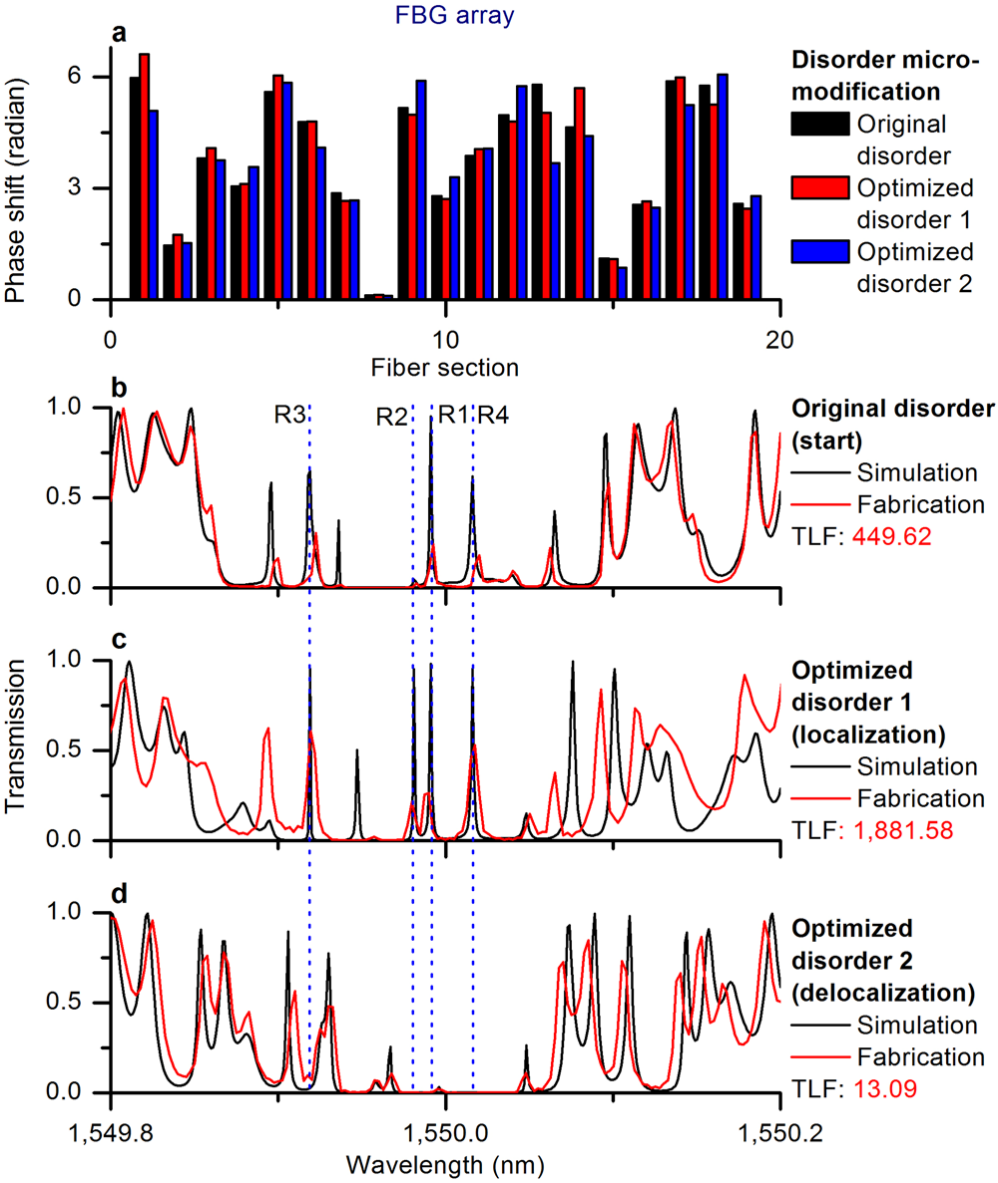
Two-way resonance optimizations between localization and delocalization of the FBG array. **(a**) Original disorder and designed (optimized) disorders. (**b**) Resonances of original disorder (simulation and fabrication) before resonance optimization. (**c**) Resonances of optimized disorder 1 (simulation and experimental fabrication) after localization resonance optimization. (**d**) Resonances of optimized disorder 2 (simulation and fabrication) after delocalization resonance optimization. TLF: total localization factor.

Here we demonstrate the efficiency of the proposed design algorithm considering an FBG array optimization. Two-way (localization and delocalization) resonance optimizations of the four variously localized resonances R1–R4 are shown in Fig. 4. We can see from Fig. 4 that by controlling the TLF via the disorder micro-modification, we have optimized both the resonant transmissions and the resonant widths of R1–R4. The localization resonance design depicted in Fig. 4c is completed after iterations and took about 4.6 minutes of numerical simulations on a standard PC, while the delocalization resonance optimization shown in Fig. 4d took only 6.3 seconds and was completed after 435 iterations. Extensive simulations prove the efficiency of the proposed one-parameter optimization technique compared to brute force direct optimization, which is not practical for systems with too many variable parameters.

The feasibility of the proposed design algorithms of the disorder micro-modification for tailoring real fiber systems was verified experimentally. Three FBG arrays with original disorder, optimized disorder 1, and optimized disorder 2 (the black, red, and blue bricks, respectively, in Fig. 4a) were manufactured (see the Methods section for details), and the corresponding transmission coefficients were measured (the red peaks in Fig. 4b–d). The red peaks in Fig. 4b–d show that all four profound localized resonances were efficiently enhanced or suppressed, demonstrating the feasibility of the proposed approach. In Fig. 4, we have optimized both the resonant transmission and spectral widths of four resonances by controlling only one parameter - the TLF. Our method might be extended to the disordered media with gain that are widely used in random lasers. For example, random lasers have been demonstrated in a randomly disordered, amplified FBG array^1, 3^, and a variety of results are available for layered medium arrays (see for example refs 8, 10, 11 and references therein). A spectral control of such random lasers can be achieved by adaptive pumping^13^. We demonstrate below that the proposed resonance design algorithm can be easily adapted to disordered mediums with gain.

### Resonance optimization of FBG array with gain

**Figure 5:**
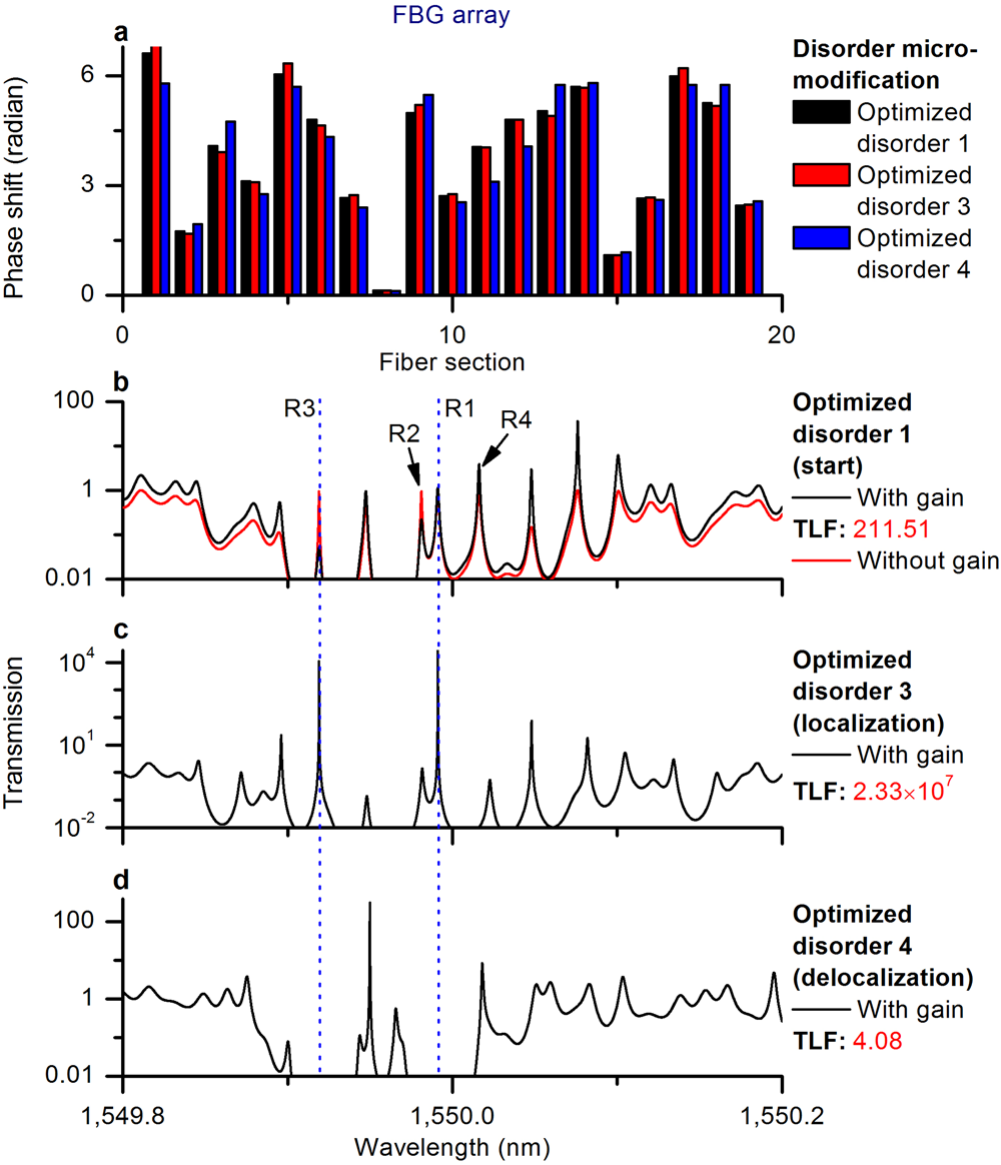
Two-way resonance optimizations between localization and delocalization of the amplified FBG array. (**a**) Optimized disorders. (**b**) Resonances of optimized disorder 1. (**c**) Resonances of optimized disorder 3. (**d**) Resonances of optimized disorder 4. TLF: total localization factor; gain rate: 20 dB/m.

**Figure 6:**
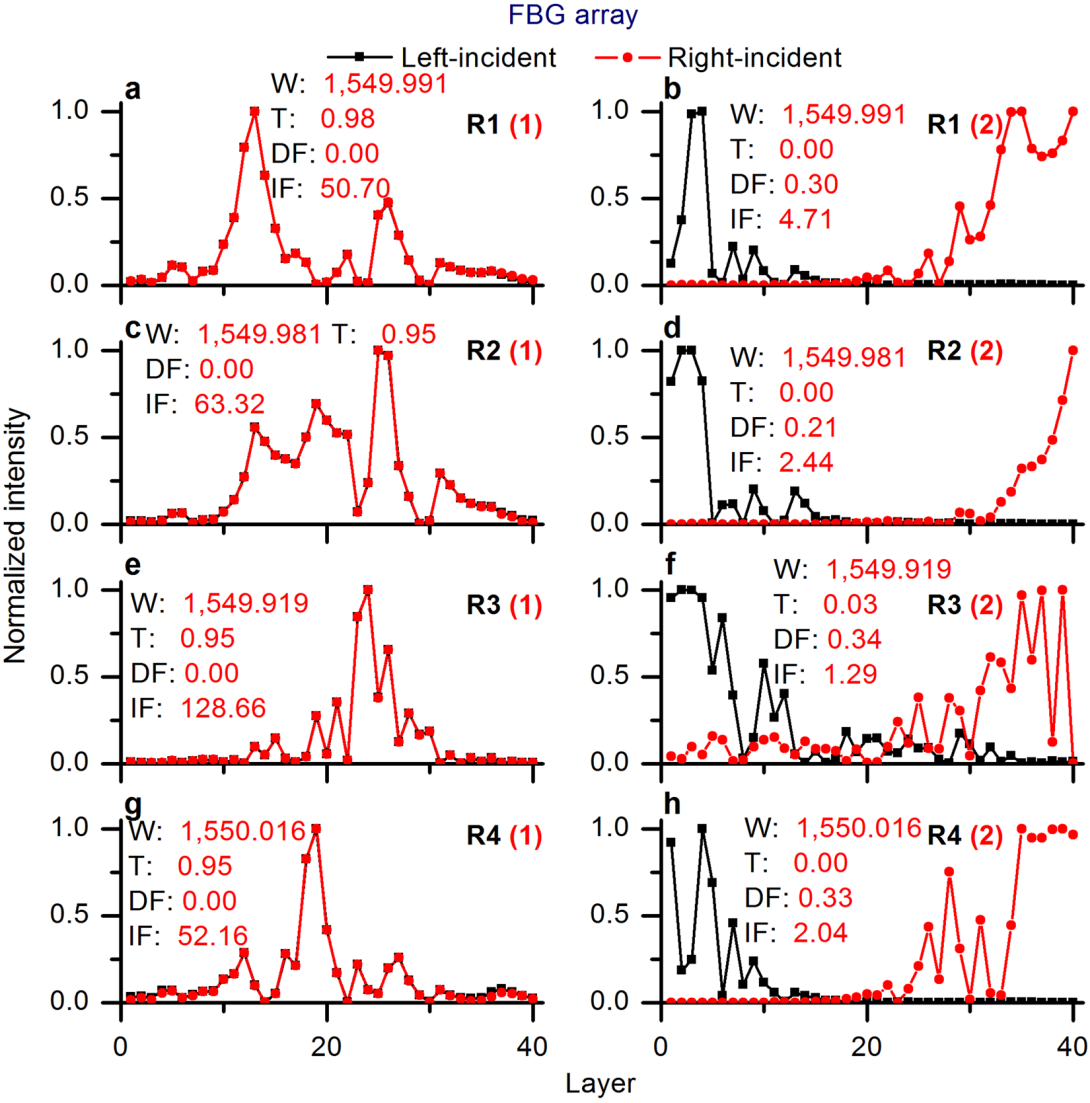
Normalized bi-directional intensity distributions in the optimized FBG arrays. (**a**) R1 (optimized disorder 1). (**b**) R1 (optimized disorder 2). (**c**) R2 (optimized disorder 1). (**d**) R2 (optimized disorder 2). (**e**) R3 (optimized disorder 1). (**f**) R3 (optimized disorder 2). (**g**) R4 (optimized disorder 1). (**h**) R4 (optimized disorder 2). W: wavelength in nm, T: transmission, DF: deviation factor, IF: intensity factor; the number in parentheses is the number of the optimized disorder.

**Figure 7:**
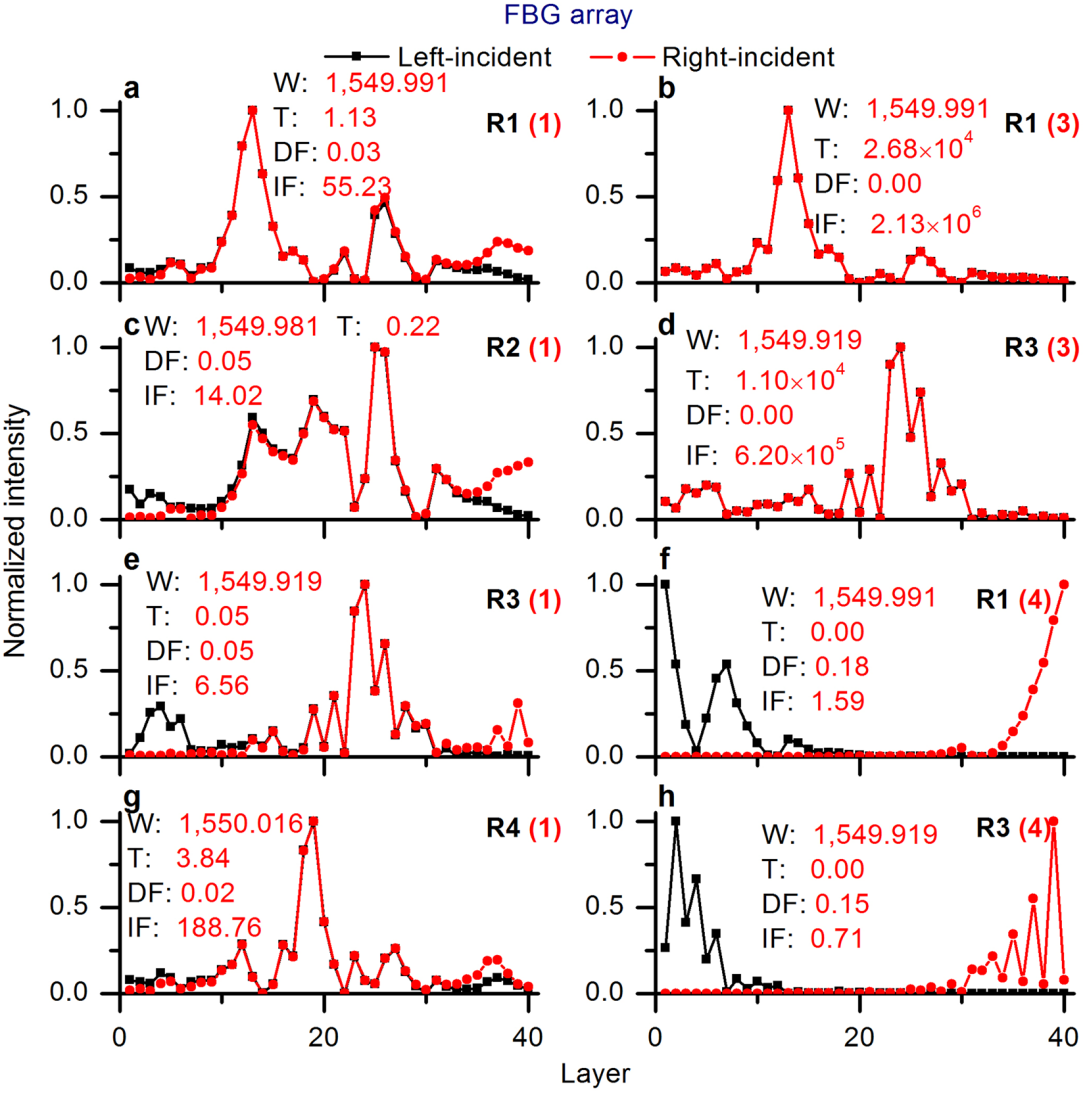
Normalized bi-directional intensity distributions in the optimized FBG arrays WITH gain. **(a**) R1 (optimized disorder 1). (**b**) R1 (optimized disorder 3). (**c**) R2 (optimized disorder 1). (**d**) R3 (optimized disorder 3). (**e**) R3 (optimized disorder 1). (**f**) R1 (optimized disorder 4). (**g**) R4 (optimized disorder 1). (**h**) R3 (optimized disorder 4). W: wavelength in nm, T: transmission, DF: deviation factor, IF: intensity factor; the number in parentheses is the number of the optimized disorder; gain rate: 20 dB/m.

We consider the FBG array with optimized disorder 1 as an example, and homogenously amplify it with a light gain of 20 dB/m. Two-way resonance optimizations between localization and delocalization of the amplified FBG array are shown in Fig. 5. We can see from Fig. 5b that light gain could lead to the suppression of some resonances (see R2 and R3 in Fig. 5b), because of the mode competition and the fact that light gain breaks the established symmetries of the bi-directional effective cavities (see Figs 6 and 7). It is seen that the localization states can be influenced both by the introduced disorder and by the pre-designed pump profile. This result is in full agreement with the observation that changes in the pump power can lead to large fluctuations of random lasing^2, 3^, and possibility of control over the emission wavelengths^13^ by adaptive pumping.

## Discussion

As shown in Figs 3 and 4, the total localization factor was increased from 449.62 to over 1,800 by modifying the original disorder (the black bricks in Fig. 4a) to the optimized sequence of the phases (disorder 1, the red bricks in Fig. 3a). Through this design optimization, all four variously localized resonances R1–R4 (the black peaks in Fig. 4b) were transformed to perfectly localized resonances (the black peaks in Fig. 4c) with close-to-unity resonant transmissions and narrow resonant widths (bi-directional high-intensity concentrations). In a similar manner, it was possible to achieve delocalization of these modes by modifying the original disorder (the black bricks in Fig. 4a) to disorder 2 (the blue bricks in Fig. 4a). Four variously localized resonances R1–R4 (the black peaks in Fig. 4b) were all changed to the perfectly delocalized resonances (the black peaks in Fig. 4c) with close-to-zero resonant transmissions and wide resonant widths (bi-directional low-intensity concentrations).

Comparing the fabricated FBG arrays with the original disordered structure (the red peaks in Fig. 4b) and the optimized case 1 (the red peaks in Fig. 4c), the measured transmission coefficients of the R1–R4 resonances increased from 0.24, 0.02, 0.30, and 0.18 to 0.26, 0.21, 0.60, and 0.53, respectively. Similarly, comparing the fabricated FBG arrays with the original disordered structure (the red peaks in Fig. 4b) and the optimized case 2 (the red peaks in Fig. 4d), the measured transmission coefficients of R1–R4 decreased from 0.24, 0.02, 0.30, and 0.18 to 0.01, 0.00, 0.07, and 0.00, respectively.

The normal FBG array with optimized disorder 1 had four perfectly localized resonances with close-to-unity resonant transmissions and narrow resonant widths (the red peaks in Fig. 5b), while the FBG array with gain with the same disorder had four variously localized resonances with fluctuating resonant transmissions and resonant widths (the black peaks in Fig. 5b). For example, R1 and R4 were only slightly amplified (R1: 0.98 up to 1.13, R4: 0.95 up to 3.84), and R2 and R3 were notably suppressed (R2: 0.95 down to 0.22, R3: 0.95 down to 0.05) with gain. R1 and R3 were strongly amplified via the disorder micro-modification (shown in Fig. 5c).

Here we propose and verify an empirical formula to describe the resonant width of the perfectly localized resonance. Figure 3 shows the normalized bi-directional intensity distributions (effective cavities) corresponding to R1–R8. For R1 and R5 with high resonant transmissions, the bi-directional effective cavities match each other perfectly (Fig. 3a and b); for R2 and R6 with low resonant transmissions (Fig. 3c and d) they deviate from each other distinctively, and for R3 and R4, and R7 and R8, with medium resonant transmissions (Fig. 3e–h) they deviate from each other to a certain extent. Thus, there is a clear link between the symmetry of the bi-directional effective cavities and the resonant transmission. The symmetry of the bi-directional effective cavities itself cannot fully describe the individual resonance. What we can see from Figs 1, 2 and 3 is that there is a relationship between the width of the resonance and the bi-directional high-intensity concentrations. For example, R3 and R4, and R7 and R8, have similar resonant transmissions: R4 has a narrower resonant width and higher bi-directional intensity concentrations than R3, and R7 has a narrower resonant width and higher bi-directional intensity concentrations than R8. Therefore, we could assume that the resonant width is inversely proportional to the bi-directional high-intensity concentrations. We introduce an empirical formula to describe the spectral width of the perfectly localized resonance:

$$F(\lambda )=\frac{{\rm{\Delta }}{\lambda }_{Bragg}}{{\beta }_{int}(\lambda )}\exp (\frac{\lambda -{\lambda }_{0}}{{\rm{\Delta }}{\lambda }_{Bragg}})$$

where *F*(*λ*) is the FWHM (full width at half maximum) of the resonance, Δ*λ*_*Bragg*_ is the width of the Bragg reflection band of the periodic FBG array, and *β*_*int*_(*λ*) is the IF. The first term $\frac{{\rm{\Delta }}{\lambda }_{Bragg}}{{\beta }_{int}(\lambda )}$ means that the resonance width is proportional to the width of the Bragg reflection band, and inversely proportional to the IF, while the term ${\rm{e}}{\rm{x}}{\rm{p}}(\frac{\lambda -{\lambda }_{0}}{{\rm{\Delta }}{\lambda }_{Bragg}})$ measures the influence of the resonance offset from the central wavelength. The four resonances in the FBG array with optimized disorder 1 are perfectly localized (Δ*λ*_*Bragg*_ = 90 pm); the FWHMs of R1–R4 obtained via numerical simulation are 1.2, 0.7, 0.4, and 1.5 pm, while those obtained with this empirical formula are 1.6, 1.2, 0.3, and 2.1 pm. The results show a good match between the numerical simulations and the presented analytical formula. Further simulations show that this empirical formula is also applicable for describing the resonant width of the layered medium.

Additional two design examples have been studied to validate the applicability of the empirical formula to the spectral width of the resonances. We considered two resonances at the wavelengths 1,549.98 nm and 1,550.02 nm. In one design, the total localization factor was increased from 4.19 to 7,044.92 following optimization of the periodic FBG array, whereas for the second design, it increased from 4.19 to 6,505.52. For design 1, the FWHMs of the two resonances found via numerical simulation were 0.36 and 0.15 pm, and those obtained via the empirical formula were 0.41 and 0.16 pm; for design 2, the FWHMs of the two resonances calculated numerically were 0.16 and 0.38 pm, and those given by the empirical formula were 0.14 and 0.46 pm. The results show that the numerical simulations and the empirical formula calculations match quite well.

We applied the proposed method to a random medium with gain, and considered two designs with the gain rate of 4.3 dB/m. The two resonances selected for optimization were at wavelengths of 1,549.98 nm and 1,550.02 nm. The total localization factor was increased from 4.22 to when changing the periodic FBG array to design 3, and was increased from 4.22 to when changing the initial FBG array to design 4. It was observed that the resonantly transmitted lasing intensity (described by the transmission) and the high-intensity concentrations (described by the IF) are extremely sensitive to the symmetry of the bi-directional effective cavities (described by the DF). For both designs, the IFs of the two resonances increased about 150 and 26 times from 74.86 and 220.50 to 1.23 × 10^4^ and 5.84 × 10^3^, while the transmissions of the two resonances increased by two orders from 1.17 × 10^4^ and 6.47 × 10^4^ to 1.77 × 10^6^ and 1.7 × 10^6^, respectively.

## Conclusion

We have proposed and demonstrated a relatively simple new method for the design or optimization of the properties of resonant transmission, including spectral widths of multiple resonances, providing a tool for randomness-on-demand design in 1D structures. The methods and conclusions presented in this work are readily applicable to FBG arrays, layered mediums, and various kinds of disordered structures. For instance, two-dimensional (2D) disordered photonic crystals could also be characterized by resonant transmission peaks accompanied with high-intensity concentrations^21, 22^. Thus, the optimization approach introduced and demonstrated in this work can be modified and applied to resonance optimization in 2D photonic crystals. Our method paves the way for the control and manipulation of high-intensity field concentration and spatial resonance localization, which can benefit various research fields via uses ranging from speckle-free imaging to photon-matter interaction and random lasing. Furthermore, quantitative resonance optimization means that novel localization-based devices are feasible, such as polychromatic filters and polychromatic lasers.

## Methods

### Calculations of the transmission coefficients and spatial intensity distributions

The overall transmission *T* and overall reflection *R* for both FBG arrays and layered mediums are determined as *T* = |*t*|^2^ and *R* = |*r*|^2^, respectively, where *t* is the complex transmission coefficient and *r* is the complex reflection coefficient, which can be found as

$$\{\begin{array}{rcl}r & = & -\,{M}_{21}/{M}_{22}\\ t & = & \mathrm{1/}{M}_{22}\end{array}$$

The transfer matrix *M* is calculated using a well-known standard transfer matrix method^23^:

$$M={M}_{{R}_{N+1}}{M}_{{C}_{N}}\ldots {M}_{{C}_{1}}{M}_{{R}_{1}}=[\begin{array}{cc}{M}_{11} & {M}_{12}\\ {M}_{21} & {M}_{22}\end{array}]$$

Here ${M}_{{C}_{j}}$ are the transfer matrices describing propagation through the fiber sections or the medium layers, and are the same for both the FBG array and the layered medium:

$${M}_{{C}_{j}}=[\begin{array}{cc}{\rm{e}}{\rm{x}}{\rm{p}}(i{\varphi }_{j}) & 0\\ 0 & \exp (-i{\varphi }_{j})\end{array}]\mathrm{,\ }{\varphi }_{j}=\frac{2\pi {n}_{j}}{\lambda }{l}_{j},$$

where *l*_*j*_ is the length of the *j*-th layer, *λ* is the wavelength. ${M}_{{R}_{j}}$ are the transfer matrices of the *j*-th FBG layer:

$${M}_{{R}_{j}}=(\begin{array}{cc}{\rm{e}}{\rm{x}}{\rm{p}}(i\delta {\rm{\Delta }}) & 0\\ 0 & {\rm{e}}{\rm{x}}{\rm{p}}(-i\delta {\rm{\Delta }})\end{array})\times {(1-{|{\rho }_{j}|}^{2})}^{-\mathrm{1/2}}[\begin{array}{cc}1 & -\,{\rho }_{j}^{\ast }\\ -\,{\rho }_{j} & 1\end{array}],$$

where Δ is the grating length, *δ* = *β* − *β*_*B*_ is the wavenumber detuning compared to the Bragg design wavenumber; ${\rho }_{j}=-{\rm{t}}{\rm{a}}{\rm{n}}{\rm{h}}(|{q}_{j}|{\rm{\Delta }}){q}_{j}^{\ast }/|{q}_{j}|$ is the discrete reflection coefficient, and *q* = *q*_*j*_ = *q*(*j*Δ) is the coupling coefficient of the *j*-th section.

In the case of a layered medium,

$${M}_{{R}_{j}}={(1-{|{r}_{j}|}^{2})}^{-\mathrm{1/2}}[\begin{array}{cc}1 & -\,{r}_{j}\\ -\,{r}_{j} & 1\end{array}],{r}_{j}=({n}_{j-1}-{n}_{j})/({n}_{j-1}+{n}_{j}\mathrm{)}.$$

The intensity through the *k*-th layer, *I*(*k*), is defined as *I*(*k*) = |*u*(*k*) + *v*(*k*)|^2^, where *u* and *v* are the wave functions of the right- and left-propagating waves, which are calculated as

$$[\begin{array}{c}u(k)\\ v(k)\end{array}]=M(k)[\begin{array}{c}{u}_{0}\\ {v}_{0}\end{array}].$$

Here, *u*_0_ is the incident (right-propagating) wave and *v*_0_ is the counter-propagating wave, and *M*(*k*) is the product of the transfer matrices of the past *k* layers. Note that *I*_0_ = |*u*_0_ + *v*_0_|^2^ is the incident intensity. For the sake of convenience and without loss of generality, we can assume the incident light is a plane wave with unitary intensity. Hence, the fields before the first layer are given by $$[\begin{array}{c}{u}_{0}\\ {v}_{0}\end{array}]=[\begin{array}{c}1\\ {\rho }_{0}\end{array}]$$.

### Normalized bi-directional intensity distributions in the optimized FBG arrays WITH and WITHOUT gain

Figures below demonstrate normalized bi-directional intensity distributions in the optimized FBG arrays without gain (Fig. 6) and with gain (Fig. 7).

### Resonance optimization scheme via SPGD method

1. Set a current controllable parameter *ϕ* = *ϕ*_0_, a vector of the initial phase shifts of all cavities.
2. Generate a random perturbation *δ*, a randomly sampled array of numbers (uniformly sampled in the range [−10^−4^, 10^4^] in this work).
3. Measure the bipolar-perturbed total localization factors *J*_+_ = (*ϕ* + *ϕδ*) and *J*_−_ = (*ϕ* − *ϕδ*).
4. Calculate the next controllable parameter *ϕ*′ = *ϕ* + *γ*(*J*_+_ − *J*_−_)*ϕδ*, where *γ* = 1/|*J*_+_ − *J*_−_| for localization optimization and *γ* = −1/|*J*_+_ − *J*_−_| for delocalization optimization. Update the controllable parameter *ϕ* = *ϕ*′.
5. If the current total localization factor *J*(*ϕ*) is larger than a preset large value (1,800 in this work) for localization optimization, or is lower than a preset small value (15 in this work) for delocalization optimization, then stop the iteration. Otherwise, go to Step 2 and do another iteration.

### Fiber Bragg grating fabrication and factors that impact the fabrication process

The gratings were fabricated in a hydrogenated SMF 28 standard telecommunications fiber using an advanced phase-mask fabrication setup. The hydrogen loading process took 4 days at 80 °C and 200 bar. The fabrication setup used a 100 mW 244 nm Argon Ion laser with an amplitude modulator controlled by a computer and a high-resolution stage. The gratings were fabricated by moving the fiber across the beam. The beam was modulated according to the apodization profile and period change of the grating. The setup had a beam size of 330 *μ*m, and the beam was convolved with the grating profile to fabricate the grating. Gratings were designed with controlled period changes to ensure the required phase shifts were fabricated accurately. The fundamental wavelength, 1,550 nm, of the mask was used. This showed that the change of phase between sub-gratings had little effect on the match between the designed and experimental gratings, considering the size of the beam used. Each grating was created with a phase shift relative to the previous grating. The gratings were annealed at 80 °C for 3 days after fabrication before being measured with an optical vector analyzer designed by LUNA. The device’s wavelength resolution for the measurements was 2.5 pm. The considerable mismatches between the simulations (the black peaks in Fig. 4b–d) and the fabrications (the red peaks in Fig. 4b–d) occur mainly because of the measurement resolution and the fabrication error.

We examine the factors that caused this mismatch, including light absorption, fabrication error, and measurement resolution, by taking the FBG array with the original disorder as an example. Considering light absorption, the absorption rate of 2 × 10^−4^ dB/m has no observable influence on the resonances, and even when the absorption rate is theoretically enlarged to 0.2 dB/m, the resonances are only lightly suppressed. Thus, light absorption is negligible for the fabricated FBG array with a short length of 6 cm.

**Figure 8:**
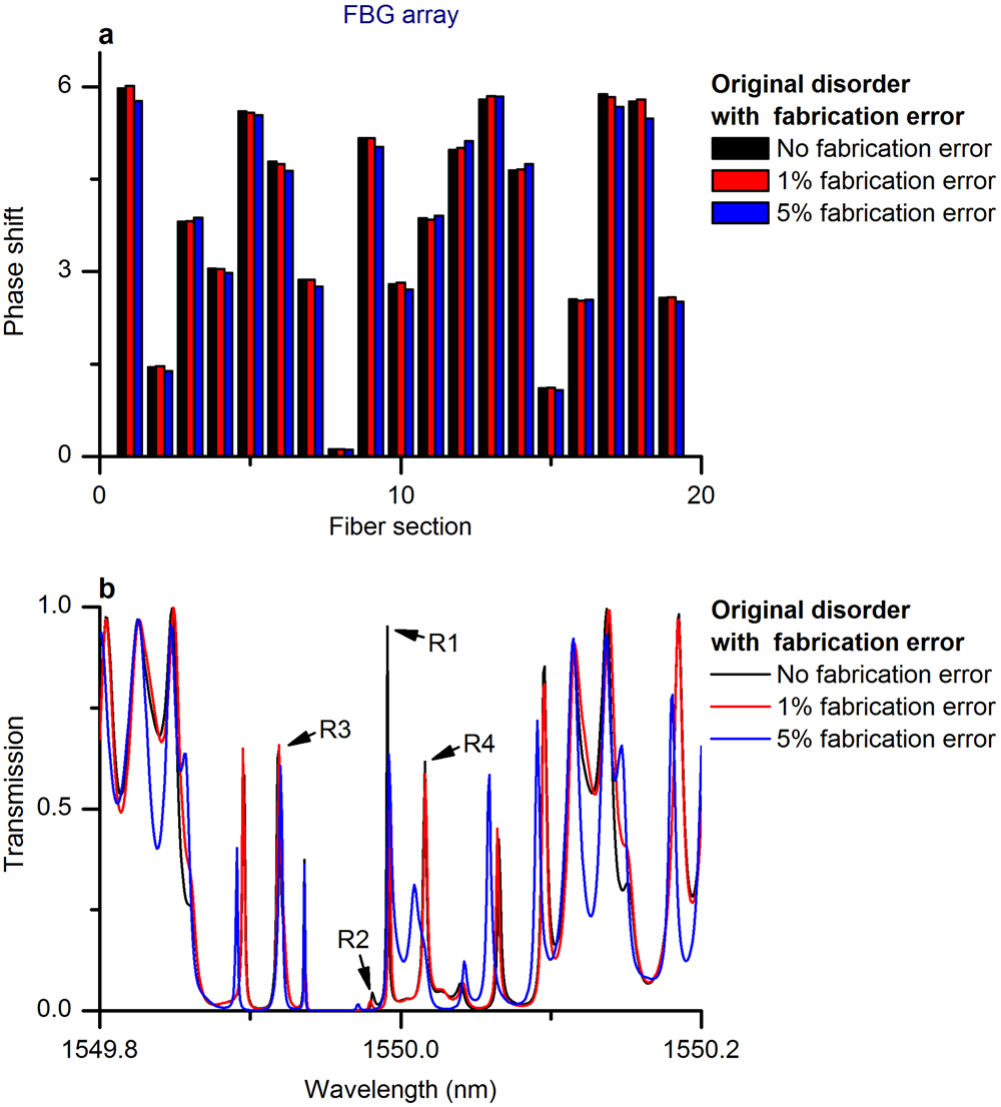
Influence of fabrication error on the FBG array with original disorder. (**a**) Fabrication error. (**b**) Error-induced resonance fluctuations and shifts.

The impact of fabrication errors is shown in Fig. 8. A fabrication error as small as up to 1% (red peaks) can affect the heights of the resonances. The effect of an up to 5% fabrication error (blue peaks) is even more visible, resulting in the shift of the spectral distribution of resonances (compared to the spectral shifting in Fig. 4). Another factor that led to the mismatch is the spectral resolution used in the simulations, 1 pm; the best possible measurement resolution was 2.5 pm. The lower measurement resolution affects the spectral averaging, especially for sharp resonances with narrow widths, and causes spectral shifting and additional fluctuations.

We can conclude that the minimal resonance mismatch between the simulation and fabrication results shown in Fig. 4 is due to fabrication errors and to the low measurement resolution.

### Comparison with the brute force optimization algorithm

The resonance optimization algorithm presented in this work is somewhat indirect. Measurable resonant transmission and the spectral widths of resonances are controlled by varying the symmetry of the left- and right-propagating light. Certainly, a direct brute force optimization of the target spectral response can be considered for the same purpose. We have performed extensive modelling using the direct resonance optimization algorithm based on the SPGD method, which steadily optimizes the current transmission spectum to a preset target transmission spectrum. We observed that, when starting from a transmission spectrum close to the target transmission spectrum, the direct resonance optimization algorithm had a relatively good performance. However, when starting from a transmission spectrum far from the target transmission spectrum, the direct resonance optimization algorithm was much slower and less efficient than the proposed method. The advantage of the proposed resonance optimization algorithm, based on TLF, is that it is independent of the starting disorder and achieves a satisfactory localization of multiple resonances more efficiently than direct optimization.

### Data availability

The datasets generated and analysed during the current study are available from the corresponding author on reasonable request.
